# Effectiveness of an Infection Control Program Among the Ysleta del Sur Pueblo in Preventing COVID-19-Related Hospitalizations and Deaths

**DOI:** 10.3390/pathogens13100913

**Published:** 2024-10-19

**Authors:** Cameron M. Torres, Victoria Aparicio, Gabriela Calzada, Ascension Mena, Charles T. Spencer

**Affiliations:** 1 Department of Biological Sciences, University of Texas at El Paso, El Paso, TX 79968, USA; cmtorres4@miners.utep.edu; 2 Speaking Rock Entertainment, Wellness Response Department, Ysleta del Sur Pueblo, El Paso, TX 79907, USAdrmena@speakingrock.com (A.M.)

**Keywords:** COVID-19, pandemic response, epidemiology, native American

## Abstract

In response to the SARS-CoV-2 pandemic, the United States declared a state of emergency and implemented large-scale shutdowns and public health initiatives to prevent overwhelming public resources. The success of these prevention methods remains unresolved as restrictions and implementation varied from national, state, and local levels. Despite national and local regulations, individual adherence to preventative guidelines presented an additional layer of variability. Cases of COVID-19 continued to rise and fall over a two-year period on a national level, despite masking recommendations, ease of testing, and availability of vaccines. The Ysleta del Sur Pueblo is a Native American tribal community and sovereign nation located in El Paso, Texas. Speaking Rock Entertainment Center is a major business operated by the tribe, employing many tribal and non-tribal members from the El Paso area. Following nationwide re-openings of non-essential businesses, Speaking Rock implemented an infection control program with strict adherence to recommendations provided by the Center for Disease Control and Prevention (CDC) and additional disease control. This response would result in a fully vaccinated workforce within the wider community of El Paso, where the vaccination rate was less than 80%. Herein, we examine the efficacy of these measures and report on the success of the program resulting in zero hospitalizations or deaths compared with rates of 1 in 250 and 1 in 40, respectively, in the surrounding community.

## 1. Introduction

SARS-CoV-2 belongs to the *Coronaviridae* family of viruses, sharing 79% of its genome identity with SARS-CoV and 50% sequence identity with Middle East respiratory syndrome coronavirus (MERS-CoV) [1,2,3], both highly virulent zoonotic coronaviruses implicated in fatal respiratory disease in humans. SARS-CoV-2 is spread from human to human via the inhalation of respiratory droplets and aerosols [4,5,6]. Entry into susceptible host cells is mediated by the binding of viral spike (S) protein with angiotensin-converting enzyme 2 (ACE2) receptors. Following this interaction, the S protein is cleaved, activating the S2 subunit which fuses the lipid bilayers of the virus and the host cell, facilitating deposition of the viral RNA genome into the cell [1,3,7,8]. The virus replicates in the upper respiratory tract initially by infecting epithelial cells in the nasal cavity. Migration deeper into the airways and lungs triggers a strong immune response and hyper-cytokinemia syndrome, referred to as a cytokine storm [9,10,11,12]. This hyper-inflammation causes cytokine release syndrome (CRS), acute respiratory distress syndrome (ARDS), and is typically the cause of death in fatal cases of COVID-19 [12,13,14].

Within a few months of gaining the attention of public health officials, the virus had spread across the globe, with over 118,000 positive cases reported in 114 countries, resulting in 4291 deaths by March 2020. The staggering scale of the outbreak and highly transmissible nature of the virus led to the World Health Organization (WHO) declaring the crisis a pandemic on 11 March 2020 [15,16,17]. Days after this announcement, individual states began implementing mandatory shutdowns of public places, schools, and non-essential businesses. During this time, the Center for Disease Control and Prevention (CDC) issued several preventative measures and guidelines including masking and social distancing recommendations. By early April 2020, the United States led the world in both confirmed cases (500,000) and deaths (18,600) attributed to COVID-19 [18,19].

The Ysleta del Sur Pueblo is a federally recognized Native American tribe (~4696 members) of the Tigua peoples, situated geographically within the 79907 and 79927 zip codes in El Paso, Texas. The Speaking Rock Entertainment Center (SREC) is a major tribal entity that provides employment opportunities for members of the pueblo as well as the wider El Paso community. Due to this shared geography, the Ysleta del Sur Pueblo and the greater El Paso region had similar exposure risks for infection with SARS-CoV-2 [20]. Following national shutdowns of non-essential businesses, SREC closed its doors mid-March 2020. It would re-open in May 2020, with reduced staffing and a rigid infection control program in place to exceed guidelines set by the Center for Disease Control and Prevention (CDC) [21,22,23]^.^ The impact of the SARS-CoV-2 epidemic within this small community was significantly controlled by the mitigation strategies implemented by SREC which, through strict enforcement, protected the community from hospitalization and death due to COVID-19.

## 2. Materials and Methods

### 2.1. Regional Infection Control Program

Within the first 6 months of the first reported positive case, El Paso would undergo several shutdowns and reopening of businesses and public places in response to ever increasing rates of infections and severe cases of COVID-19. Businesses were allowed to reopen at reduced capacity initially, with social distancing and sanitation protocols in place including barrier protection and masking recommendations. By November 2020, El Paso gained international news attention due to the pandemic overwhelming the public health system. Then, 2021 witnessed an intense political and legal battle between the state and local governments to fully reopen businesses and public places and repeal mask mandates. This controversy likewise spilled into vaccination and treatment efforts. With the politically charged nature and controversy surrounding their use, masking and vaccination recommendations were not strictly adhered to.

### 2.2. SREC Infection Control Program

As a sovereign nation, the Ysleta del Sur Pueblo had authority to enact and enforce a public health program independent of that in the surrounding city, county, and state governances. SREC has an onsite Wellness Response Department with a medical doctor and nurses available to the employees and families of the Pueblo. At the onset of the COVID-19 pandemic, the Wellness Response Department was charged with designing and instituting infection control protocols with the aim of stemming the spread of the virus. The program enacted by SREC consisted of sterilization equipment, facility upgrades, and medical surveillance to reduce the incidence and spread of SARS-CoV-2 amongst employees, families, visitors, and the community. As the facility was reopened in May 2020, commercial air purifiers and surface purifiers were installed along with sterilization tunnels (Disinfect Group) at all entrances. These tunnels consisted of a thermal monitoring system to detect elevated body temperatures, touchless hand sanitizer dispensers, and a sanitization fog chamber. All staff and patrons had to pass through these sterilization tunnels before entering the center. The entire facility was sprayed with antimicrobial solution (PurTabs Hospital Disinfectant Effervescent Tablets) three times a day. Partitions between individual stations separated patrons and mandatory mask mandates were implemented for all guests and employees.

Onsite PCR testing, following CDC guidelines, was made available to employees and their families by mid-May 2020, and the testing center was open 16 h a day, 7 days a week. This step negated the need to test at a city testing center. If any employee did test positive at an external testing center, they would be re-tested at the center to confirm the results of the previous test. Wait times for the results of PCR tests were on par with the city of El Paso, initially 2–3 days, but then were significantly lengthened, 10–12 days, as testing demand increased and ultimately decreased to 7 days turn around. Employees who tested positive were quarantined for 14 days early in pandemic response. This was later reduced to 7 days following recommendations by the CDC. As test result wait times increased due to increased testing numbers, the center transitioned to commercially available rapid antigen testing of various manufacturers in December 2020. Employees and family members testing positive with a rapid antigen test were confirmed by a PCR amplification test. The correlation between rapid antigen test and PCR test was 100% in this report.

### 2.3. Data Collection and Analysis

Employees of SREC receive a health screening when hired at the center, and medical services are available to them and their families throughout their employment. Thus, medical records are well documented and routinely monitored. The health staff additionally collected employee data relevant to the ongoing pandemic. These data consisted of household contact incidences, prior health conditions which may complicate a COVID-19-positive diagnosis, results of onsite, and external COVID-19 tests, and vaccination data. Along with the El Paso County Health Department, SREC Wellness Response Department medical staff performed contact tracing for their COVID-19-positive cases.

Data analysis for this study was performed by personnel at the University of Texas at El Paso (UTEP) not affiliated with SREC. To protect private health information, the identity of employees was anonymized, and individual sets of data were given a unique identification number. The retrospective analysis of this data was reviewed by the Institutional Review Board at UTEP and they deemed it exempt since the data UTEP personnel received were de-identified and resultant analysis would only present population data. These data were compared with publicly available data reporting the outcomes of the pandemic in the surrounding El Paso area to determine the efficacy of the respective control measures.

## 3. Results

**Figure 1:**
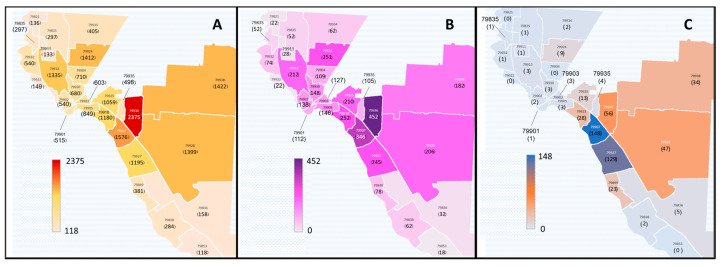
Distribution of severe COVID-19 and residence of SREC employees within El Paso County. Regions impacted by COVID-19 resulting in high incidence of hospitalizations (**A**) coincide with areas of high COVID-19-related deaths (**B**). In panel A, hospitalization rates are colored on a graduatedscale from light orange (low) to red (high). In panel B, light pink indicates low death rates; are colored on a graduated scale from light pink (low) to purple (high). (**C**) In panel C, 82% of SREC employees reside within areas of high hospitalization and death rates, as indicated by the resident density gradient color scale from light blue (few) to dark blue (many).

In west Texas, the city of El Paso and the surrounding communities in El Paso County (1015 sq mi) were severely affected by the pandemic. The population of 865,657 reside within 25 zip codes, 79821–79938. At the time of this study, El Paso County had seen 19,949 hospitalizations (Figure 1A) and 3691 deaths (Figure 1B) as a result of COVID-19, with >50% of hospitalizations and deaths from those residing within 7 zip codes: 79907, 79912, 79925, 79927, 79928, 79936, 79938 [20,21]. These regions constituted the heaviest burden of SAR-CoV-2 in the El Paso community.

While the entertainment center is located within the Pueblo, it does not employ only tribal members, with 47.04% of employees outside of the tribe (Table 1). At the time of this study, SREC employed ~520 people in a variety of positions. Collectively, the majority (82.22%) of its employees reside in the nearby 79907 and 79927 zip codes (Figure 1C). Socioeconomic indices of education, household income, employment, and access to health insurance show no significant difference between the Ysleta del Sur Pueblo and the surrounding El Paso County zip codes (*p* = 0.7812 by chi-square test, Supplementary Table S1) [24].

In addition, SREC serves the entire El Paso metropolitan area, including all El Paso County, southern New Mexico, and Ciudad Juarez, Mexico, providing a high chance of exposure to infectious diseases to employees. This was of particular concern during the SARS-CoV-2 pandemic in 2020–2021. As the Ysleta del Sur Pueblo are a self-governing body located within the El Paso County area with a large public interaction, this provided a unique opportunity to directly compare two sets of epidemiological control measures for a comingled group of individuals in an isolated geographic space.

The public health burden of COVID-19 was quite severe on the El Paso community, with 38% of the population testing positive for SARS-CoV-2 infection [20,21]. Of those, 19,949 cases resulted in hospitalizations with 3698 deaths (Table 2). Conversely, SREC reported 320 positive COVID-19 cases, representing 61% of their employees. Despite this significantly higher infection rate (Table 2, * *p*-value < 0.0001 by Fisher’s exact test) none resulted in severe disease requiring hospitalization and there were no deaths attributed to COVID-19 (Table 2, * *p*-value < 0.0001 by Fisher’s exact test).

**Figure 2:**
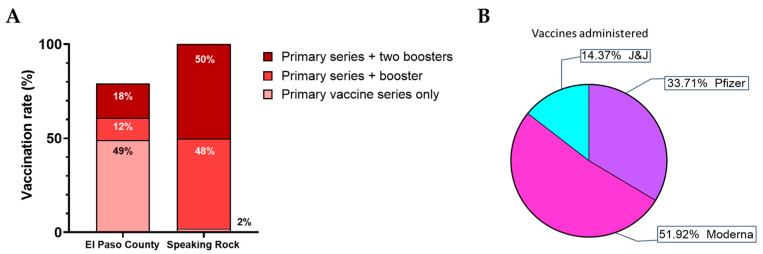
Vaccinations of SREC and El Paso County residents. Public Health officials reported vaccination rates in El Paso County compared with SREC vaccination rates (**A**). SREC had overall significantly higher levels of vaccination (*p*-value < 0.0001 by chi-square test). Primary vaccine series consisted of a single dose of the Johnson and Johnson (J&J) protein vaccine or two doses of the Moderna or Pfizer mRNA vaccines. Subsequently, booster vaccines were released against novel variants that had arisen. (**B**) Percentages of the primary vaccine series received by SREC employees by developer.

During this time period, El Paso County health officials reported just under 80% of the El Paso County population received at least the initial immunization against SARS-CoV-2, despite CDC recommendations, with 49% of the populace receiving only the primary vaccine series and 31% receiving at least one booster (Figure 2A) [20,21]. By contrast, the SREC Wellness Response Department ensured that all medically qualified employees were vaccinated against SARS-CoV-2 as effective vaccines became available in January 2021 and subsequently with the first then second booster providing protection against novel variants. Furthermore, 100% of SREC employees had been vaccinated against SARS-CoV-2 infection, with 98.5% receiving at least one booster immunization (Figure 2A) [25]. The distribution of vaccine manufacturers varied, with 85% of employees receiving mRNA-based vaccines and their boosters (Figure 2B). By June 2021, all staff were fully vaccinated, and all new hires would receive their first doses upon hiring. These high vaccination rates likely contributed significantly to the lack of severe COVID-19 with SREC.

Employee health data were collected by the SREC Wellness Department, including infection rates, household exposures, vaccination status, and health conditions throughout the pandemic. The frequency of comorbidities associated with increased COVID-19 severity were similar amongst all staff, COVID-positive employees, and COVID-19-related deaths in the general El Paso County (Table 3). Because there were no deaths among SREC employees, comorbidities among El Paso County (EP) COVID-19-related deaths were compared with SREC COVID-19-positive cases. Incidences of chronic lung disease, diabetes and pre-diabetes, and cardiovascular disease showed no significant difference between these populations (Fisher’s exact test). In contrast, hypertension rates were significantly more frequent in SREC employees compared with the general population (* *p*-value 0.0003 by Fisher’s exact test). Despite this, there were no associated hospitalizations or deaths associated with COVID-19.

**Figure 3:**
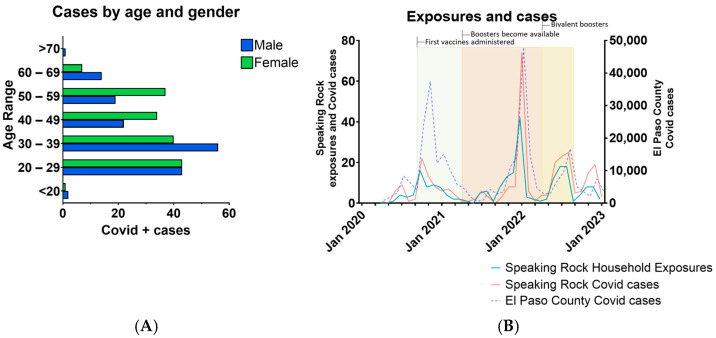
Demographics and contact tracing of COVID-19-positive cases at SREC. The majority of SREC positive cases (**A**) were among employees 39 and under, with males slightly higher than females. Exposure rates at SREC corresponded with rates of exposure in El Paso County (**B**). Incidence of disease in SREC household contacts (blue line) trended just prior to the incidence of disease in SREC employees (red line).

A critical aspect of the COVID-19 mitigation program was the availability of testing services to both employees and their family members as well as the tracking tracing of exposures to COVID-19-positive cases. The incidence of disease among employees indicated that younger males (aged 30–39 and 20–29) had the highest incidence of disease, which declined rapidly in older males (Figure 3A). Interestingly, among female employees the disease incidence was similar across a wide age range (20–59).

COVID-positivity of both SREC employees and household contacts generally correlated with the greater El Paso County community transmission rates (Figure 3B). Of notable exception was December 2020 to February 2021, during which there were disproportionately lower numbers of COVID-19-positive cases among SREC employees and their families. This suggests the effective control of viral spread by the prevention strategies enacted early in the pandemic prior to introduction of vaccination. Indeed, detailed analysis of employee and household contact tracing revealed that COVID-19 positivity was first observed in household contacts. This was then followed by a concomitant rise in SREC employee COVID-positive cases. These data suggest that the majority of COVID-19 cases at SREC occurred due to acquisition by household contacts who then transmitted the infection to SREC employees. This suggests there was limited to no transmission of SARS-CoV-2 within SREC, providing additional evidence of the success of their prevention and control activities.

## 4. Discussion

As a retrospective study of the outcomes of a mitigation and control program rapidly implemented during the pandemic crisis, this study is limited in only being able to report data collected on a routine basis during the pandemic. Specific testing of procedures, patient samples, etc., thus cannot be retroactively performed or re-analyzed. Consequently, any conclusions drawn from this report must be evaluated as a summary of data collected and detailing of practices enacted in response to a public health crisis.

The similarity of exposure risk between these two groups is important for programmatic comparison between the outcomes of SREC and El Paso County. The Ysleta del Sur Pueblo resides entirely within the El Paso County borders and SREC draws employees and patrons from across the region. This comingling should normalize exposure risk across the two groups. Likewise, the two groups had similar COVID-19 comorbidity risk factors, such as hypertension, cardiovascular disease, lung disease, tobacco use, obesity, and diabetes [26,27]. Uniquely, hypertension was specifically elevated in the SREC contacts compared with the general El Paso County residents. However, despite this increase there have been no hospitalizations or deaths from COVID-19 among SREC employees. Due to the pathology of COVID-19, it is likely that while hypertension may contribute to exacerbation of disease, it is less likely to be directly involved in the initial infection.

The impact of SARS-CoV-2 has been felt across the globe over the past 3 years. Communities have been differently affected by the pandemic and the associated economic burden. Despite the advances in vaccine development and medical technology, vaccine hesitancy, social disparities, mistrust, and resistance to medical science have exacerbated the toll this virus has had on human health [28,29]. A lack of cooperation by the general populace for greater public health was apparent from the SARS-CoV-2 public health crisis. In this report, we highlight a program that effectively prevented morbidity and mortality in a community with a high burden of COVID-19. This program exceeded recommendations established by the Center for Disease Control and Prevention and, through that program, effectively reduced the spread of disease amongst its population. An important aspect of this localized control program was the social adherence to control guidelines, availability of testing, and contact tracing programs.

This resulted in a 100% vaccination rate for the primary series, with the initial booster made mandatory regardless of which initial vaccine was received. Primary and secondary doses as well as boosters were administered on an employee’s last day of work in a given week to ensure they would be home should any side effects occur. Once bivalent boosters were developed, which provide a broad spectrum of protection across a range of COVID-19 variants, they were made available to staff [25]. At the present time, nearly 50% (well above national rates) of employees had received the bivalent booster. With the CDC trending towards recommending yearly booster vaccines [25,30], it is likely that SARS-CoV-2 vaccine protection will continue to remain at the forefront to combating COVID-19 disease.

The control and mitigation program implemented by SREC, including mandatory masking, sterilization tunnels, surface, and air sanitation, was ultimately effective at preventing hospitalization and the death of individuals under their purview. While this is not overly different from other commercial mitigation techniques in response to the pandemic, with the exception of the sterilization tunnels, public acceptance was different. Due to the sovereign governance of the Ysleta del Sur Pueblo, SREC was able to implement these strategies with an extremely high compliance rate. The success of this program underscores the importance of intensive infectious disease control in public health programs and policies and highlights the need for cooperation among the populace. These observations encourage widespread access to healthcare and infection monitoring in reducing the community burden of severe disease and increased public participation in Public Health Official’s mitigation strategies.

## Figures and Tables

**Table 1:** Speaking Rock Entertainment Center employees.

	Male	Female	Total	*p*-Value
Tribal members	27.30%	25.60%	52.90%	0.793
Non-tribal members	24.80%	22.30%	47.10%	

**Table 2:** COVID cases in El Paso County and SREC.

	El Paso County	SREC	*p*-Value
Population	837,654	523	
COVID positive cases	321,827 (38.4%)	320 (61.2%)	<0.0001 *
Hospitalizations	19,949 (6.2%)	0 (0%)	<0.0001 *
Deaths	3698 (1.15%)	0 (0%)	0.0058 *
Vaccination rate	79.26%	98.47%	<0.0001 *

* *p*-value < 0.001 by Fisher’s exact test.

**Table 3:** Comorbidities among SREC employees and El Paso COVID-related deaths.

	EP COVID-19 Deaths (3698)	SREC COVID-19 Positive (320)	*p*-Value
Hypertension	10.00%	16.90%	0.0003 *
Diabetic/pre-diabetic	11.00%	14.70%	0.0528
Chronic lung disease	6.00%	8.77%	0.054
Cardiovascular disease	6.00%	4.70%	0.3876

* *p*-value = 0.003 by Fisher’s exact test.

## Data Availability

The data presented in this study are available on request from the corresponding author due to the presence of private health information.

## Supplementary Materials

The following supporting information can be downloaded at https://www.mdpi.com/article/10.3390/pathogens13100913/s1, Table S1: Regional Socioeconomic Demographics.
